# Molecular Detection of Carbapenemase-Encoding Genes in Multidrug-Resistant *Acinetobacter baumannii* Clinical Isolates in South Africa

**DOI:** 10.1155/2020/7380740

**Published:** 2020-06-13

**Authors:** Yaw Adjei Anane, Teke Apalata, Sandeep Vasaikar, Grace Emily Okuthe, Sandile Songca

**Affiliations:** ^1^Division of Medical Microbiology, Department of Laboratory Medicine & Pathology, Faculty of Health Sciences, Walter Sisulu University, Private Bag: X1, Mthatha 5117, Eastern Cape Province, South Africa; ^2^Division of Medical Microbiology, National Health Laboratory Services (NHLS), Nelson Mandela Central Hospital, Mthatha 5100, South Africa; ^3^Department of Biological & Environmental Sciences, Walter Sisulu University, Private Bag: X1, Mthatha—5117, Eastern Cape Province, South Africa; ^4^School of Chemistry and Physics, College of Agriculture Engineering and Science, University of KwaZulu-Natal, 2nd Floor, Francis Stock Building, Howard College Campus, UKZN, Durban 4041, South Africa

## Abstract

**Introduction:**

Carbapenem-resistant *Acinetobacter baumannii* has been responsible for an increasing number of hospital-acquired infections globally. The study investigated the prevalence of carbapenemase-encoding genes in clinical multidrug-resistant *A. baumannii* strains.

**Materials and Methods:**

A total of 100 nonduplicate multidrug-resistant *A. baumannii* strains were cultured from clinical samples obtained from healthcare facilities in the O. R. Tambo district. The strains were confirmed by detecting the intrinsic *bla*_OXA-51-like_ gene. Antimicrobial susceptibility testing was performed by VITEK^®^ 2 and autoSCAN-4 systems. The MIC of imipenem and meropenem was rechecked by E-test. Colistin MIC was confirmed by the broth microdilution method. Real-time PCR was performed to investigate the presence of carbapenemase-encoding genes.

**Results:**

Most strains showed high resistance rates (>80%) to the antibiotics tested. Resistance to amikacin, tetracycline, and tigecycline were 50%, 64%, and 48%, respectively. All strains were fully susceptible to colistin. The *bla*_OXA-51-like_ was detected in all strains whilst *bla*_OXA-23-like_, *bla*_OXA-58-like_, *bla*_OXA-24-like_, *bla*_IMP-1_, *bla*_VIM_, and *bla*_NDM-1_ were found in 70%, 8%, 5%, 4%, 3%, and 2% of strains, respectively. None of the tested strains harboured the genes *bla*_SIM_ and *bla*_AmpC_. The coexistence of *bla*_OXA-23-like_, and *bla*_IMP-1_ or *bla*_OXA-58-like_ was detected in 1% and 2% strains, respectively. A distinct feature of our findings was the coharbouring of the genes *bla*_OXA-23-like_, *bla*_OXA-58-like,_ and *bla*_IMP-1_ in 2% strains, and this is the first report in the Eastern Cape Province, South Africa. The *intI*1 was carried in 80% of tested strains whilst IS*Aba*1/*bla*_OXA-51-like_ and IS*Aba*1/*bla*_OXA-23-like_ were detected in 15% and 40% of the strains, respectively. The detection of *bla*_OXA-23-like_, IS*Aba*1/*bla*_OXA-51-like_, IS*Aba*1/*bla*_OXA-23-like_, and *bla*_OXA-23-like_, *bla*_OXA-58-like_, and *bla*_IMP-1_ carbapenemases in strains had a significant effect on both imipenem and meropenem MICs.

**Conclusions:**

Results showed a high level of oxacillinases producing *A. baumannii* circulating in our study setting, highlighting the need for local molecular surveillance to inform appropriate management and prevention strategies.

## 1. Introduction

*Acinetobacter baumannii*, an opportunistic Gram-negative bacterium, has become a predominant cause of healthcare-associated infections worldwide [1]. This microorganism has emerged to become a major concern for clinicians worldwide causing infections such as septicaemia, meningitis, endocarditis, ventilator associated pneumonia, burns, urinary tract infections, surgical site infections, and wound infections in hospitals due to its intrinsic as well as its remarkable propensity to rapidly acquire resistance determinants to a wide range of antibacterial agents [2, 3]. The World Health Organization (WHO) lists *A. baumannii* among critical antibiotic-resistant “priority pathogens,” highlighting its serious threats to public health [4]. Risk factors for *A. baumannii* infections include immune suppression, burns, trauma, mechanical ventilation, catheters, invasive medical procedures, previous antibiotic treatment, an extended hospital stay (>90 days), and underlying diseases such as diabetes [5].

Carbapenem has been the drug of choice used to treat MDR *A. baumannii* infections over the past decade because they are highly efficacious and have low toxicity [6]. However, the resistance rate against carbapenem has increased dramatically due to its frequent application in recent years and is mainly mediated by the production of carbapenem-hydrolysing enzymes [7]. The most common mechanism of carbapenem resistance *in A. baumannii* is the production of *β*-lactamases, including enzymes of Ambler Classes D OXA-type carbapenemases consisting of *bla*_OXA–23-like_ (OXA–23, OXA–27 and OXA–49), *bla*_OXA–40-like_, (OXA–24/40, OXA–25, OXA–26 and OXA–72), *bla*_OXA–58-like_ (OXA–58 and OXA–96), *bla*_OXA–143-like_ and *bla*_OXA–51-like_ enzymes, and B Metallo- *β* -lactamases (MBLs) mainly *bla*_VIM_, *bla*_IMP-1_, *bla*_NDM-1_, *bla*_SPM_, *bla*_GIM_, and *bla*_SIM_ types [8]. Among the multiple types of MBL genes described throughout the world, the *bla*_IMP-1_, *bla*_VIM_, and *bla*_SIM_ types are the most common [9]. The upstream of OXA-type class D carbapenemases in *Acinetobacter* is often associated with insertion sequence (IS), of which IS*Aba*1 is the most commonly detected. IS*Aba*1 and other IS may modulate the expression and transfer of OXA-type carbapenemase genes [10]. Integrons have been demonstrated to play a major role in the dissemination of multidrug-resistance among Gram-negative bacteria through the capture and expression of resistant genes embedded within cassettes [11].

Studies in Africa and the Gulf Cooperation Council (e.g., Saudi Arabia) have reported increasing carbapenem resistance among *A. baumannii* isolates [12–14]. Since nosocomial *A. baumannii* infection has generated an abundance of therapeutic obstacles for the treatment of hospitalized patients in South Africa, as well as other countries, the knowledge regarding the prevalence level of antibiotic resistance genes and the resistance pattern of this bacterium to the initial antibiotics is required to control, prevent, and cure *A. baumannii* driven infections. Therefore, the aim of the current study was to investigate the rates of carbapenem resistance as well as to detect the frequency of carbapenemase-encoding genes in MDR *A. baumannii* isolates obtained from hospitalized patients receiving treatment in healthcare facilities in O.R. Tambo district in the rural Eastern Cape Province of South Africa.

## 2. Materials and Methods

### 2.1. Study Design and Settings

This was a prospective cross-sectional study conducted at healthcare facilities in the OR Tambo district municipality, Eastern Cape, South Africa. The municipality is formed by five local municipalities (King Sabata Dalindyebo, Nyandeni, Mhlontlo, Port St Johns, and Ingquza Hill) with an estimated total population of 1,760,389. Healthcare services are delivered by 1 Academic Central Hospital, 1 Regional Hospital, 12 District Hospitals; 11 Community Health Centers, 49 clinics, 52 Health Posts, and 15 mobiles. Patients' clinical samples collected from all these facilities are sent for culture and susceptibility testing in the Department of Medical Microbiology at the National Health Laboratory Services (NHLS), located in the Nelson Mandela Central Hospital in Mthatha, Eastern Cape. Clinical samples from those various hospitals and clinics were sent as part of the patients' routine standard of care.

### 2.2. Data Collection, Bacterial Identification, and Storage of *A. baumannii* Isolates

From August 2016 to July 2017, a total of 100 nonduplicate nonconsecutive MDR *A. baumannii* strains were collected arbitrarily from blood, pus, urine, sputa, catheter tips, tracheal aspirates, and cerebrospinal fluid of patients from the Microbiology Laboratory at the National Health Laboratory Services (NHLS) at NMAH, Mthatha (see Supplementary Materials). The strains were preliminarily speciated phenotypically using standard microbiological procedures: the VITEK® 2 automated machine with Gram-negative identification (GNI) cards (BioMérieux, France) and the MicroScan autoSCAN-4 System (Dade Behring Inc., Deerfield, IL) with Gram-negative ID type 2 panel. A high percentage (≤95%) was utilized as the acceptance criterion for identification by the MicroScan autoSCAN system. Suspected colonies were also further verified using the *Acinetobacter* specific primer set Ac436F and Ac676r to amplify the 16S rRNA gene. Confirmation of *A. baumannii* strains was carried out by polymerase chain reaction analysis of the presence of inherent *bla*_OXA-51-like_ genes. All of the strains were stored at −80°C in skim milk with 15% glycerol until further use.

### 2.3. Antimicrobial Susceptibility Testing

A total of 18 clinically relevant antibiotics were tested using MicroScan autoSCAN-4 Gram-negative MIC panel, and results were analyzed and interpreted according to the recommended clinical breakpoints given in the clinical and laboratory standard institutes (CLSI) guidelines v27 [15]. These antibiotics were amikacin (AMK), ampicillin/sulbactam (AMS), cefepime (FEP), cefotaxime (CTX), ceftazidime (CAZ), ceftriaxone (CRO), ciprofloxacin (CIP), gentamicin (GEN), imipenem (IMP), meropenem (MEM), levofloxacin (LVX), tetracycline (TEC), tobramycin (TOB), trimethoprim/sulfamethoxazole (SXT), piperacillin/tazobactam (TZP), piperacillin (PRL), colistin (CST), and tigecycline (TGC), all from Beckman Coulter, South Africa. Determination of MICs to imipenem and meropenem rechecked by E-test minimum inhibitory concentration method using E-test strips as per the manufacturer's guidelines (BioMérieux, France) on Mueller-Hinton agar plates and CLSI breakpoints (strains displaying MICs ≥8 *µ*g/mL for imipenem and meropenem were considered resistant). Quality control strains used in antimicrobial susceptibility testing are *Escherichia coli* ATCC #25922, *Staphylococcus aureus ATCC* 29213, and *Pseudomonas aeruginosa* ATCC #27853 [16]. Colistin susceptibility testing was confirmed using the broth microdilution method according to the CLSI and by ComASP™ Colistin, Liofilchem^®^ (REF. 75001), according to the manufacturer's recommendations. *Escherichia coli* NCTC 13846 (*mcr-*1 positive) was used as a positive control for ComASP™ Colistin test. The United States Food and Drug Administration (US-FDA) approved breakpoints for members of the family *Enterobacteriaceae* were used as interpretative criteria for tigecycline (http://www.fda.org.uk/sitemap.aspx). Nonsusceptibility was defined as a combination of resistance and intermediate resistance. MDR *A. baumannii* isolates were defined as acquired nonsusceptibility to at least one agent in three or more antimicrobial categories [17]. All results were within quality control ranges.

### 2.4. Detection of Carbapenem-Resistant Genes

Genomic DNA from an overnight culture on tryptic soy broth of *A. baumannii* was extracted using the MagNaPure Compact^®^ nucleic acid isolation kit I (ref. no. 03730964001, Roche Diagnostics, Mannheim, Germany) according to the manufacturer's protocol. A final elution volume of 200 *µ*L of pure *A. baumannii* DNA was used. 2 *µ*L of the DNA extract was used for each PCR analysis. The extracted DNA was stored at −80°C until required for further analysis. All MDR isolates were systematically screened by LightCycler 2.0 real-time PCR (Roche Diagnostics) instrument targeting some of the epidemiologically most relevant resistant genes. The presence of the genes encoding Amber class D Serine-Carbapenemase genes (*bla*_OXA-51-like_, *bla*_OXA-23-like_, *bla*_OXA-24-like_, and *bla*_OXA-58-like_), Ambler class B Metallo-*β*-lactamases (MBLs) (*bla*_IMP-1_, *bla*_VIM_, *bla*_SIM_, and *bla*_NDM-1_), and Ambler class C *bla*_AmpC_ were assessed. MDR *A. baumannii* strains were subsequently screened for the presence or absence of IS element IS*Aba*1 [18] and class 1 integrons by real-time PCR [19]. To determine the overexpression of *bla*_OXA-23-like_ and *bla*_OXA-51-like_ families due to the upstream adjacent IS*Aba*1, PCR was firstly performed for IS*Aba*1, and in cases where *bla*_OXA-like_ is present, a combination of forward primer of IS*Aba*1 and reverse primer of *bla*_OXA-like_ family was further used. Appropriate positive and negative controls were run simultaneously (Table 1). The primers used for PCR amplification of the carbapenemase genes are listed in Table 2.

### 2.5. Statistical Analyses

Data analyses were performed using the Statistical Package for Social Sciences Statistics for Windows software package version 23.0 (IBM, Armonk, NY). Results were expressed using frequency and percentages for qualitative variables and as mean (standard deviation) or median (interquartile range: IQR) for quantitative variables. The association between *A. baumannii* and drug resistance was analyzed by the chi-square test. All *P* values were two-sided. A *P*-value <0.05 was considered statistically significant.

### 2.6. Ethical Considerations

Ethical approval for this project was obtained from the Walter Sisulu University Research Ethics and Biosafety Committee (Reference number: 019/2016). No written informed consent was required because each MDR *A. baumannii* isolate obtained was delinked with the patient's personal information. Also authors had no direct interactions with patients from whom these isolates were isolated.

## 3. Results

### 3.1. Demographic Characteristics of the Study Population

The demographic characteristics of the patients with *A. baumannii* infection analyzed in our study showed a slight female preponderance of 47% males versus 53% females). The age of the overall patients ranged between 1 day and 91 years (mean 44.4 years, SD ± 36.3 years). *A. baumannii* were isolated highest from tracheal aspirate (32%) and lowest from cerebrospinal fluid (2%). *A. baumannii* was most prevalent in the ICU wards 41% followed by neonatal ward 22%, surgical ward 15%, medical ward 13%, and general ward 9%. *A. baumannii* was isolated from various age groups but was the most prevalent in age group 0–9 years (Supplementary Materials).

### 3.2. Antimicrobial Susceptibility Testing

**Figure 1:**
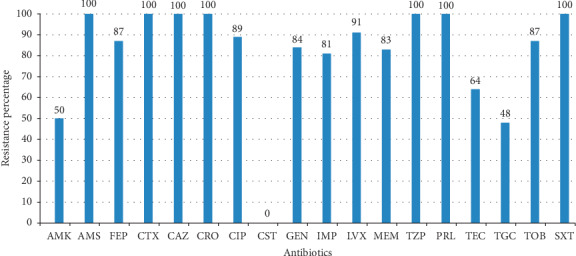
Antimicrobial resistance profile of 100 *A. baumannii* isolates. IMP: imipenem; MEM: meropenem; FEP: cefepime; CAZ: ceftazidime; CTX: cefotaxime; CRO: ceftriaxone; AMS: ampicillin/sulbactam; PRL: piperacillin; TZP: piperacillin/tazobactam; AMK: amikacin; TOB: tobramycin; GEN: gentamicin; TEC: tetracycline; CIP: ciprofloxacin; LVX: levofloxacin; SXT: trimethoprim-sulfamethoxazole; TGC: tigecycline; CST: colistin.

Generally, the results for antimicrobial susceptibility testing for the 100 MDR *A. baumannii* revealed that *A. baumannii* strains showed a high resistance rate to most of the commonly used antibiotics tested. Overall, ≥81% of all the strains were resistant to extended-spectrum cephalosporins, fluoroquinolones, and carbapenems antibiotics. Resistance to aminoglycosides ranges from 50% to 87% whilst 48% of strains were resistant to tigecycline. All strains were susceptible to colistin. Findings of the antimicrobial susceptibility patterns of all *A. baumannii* strains in this study are shown in Figure 1.

### 3.3. PCR Results of Carbapenemase-Encoding Genes

**Figure 2:**
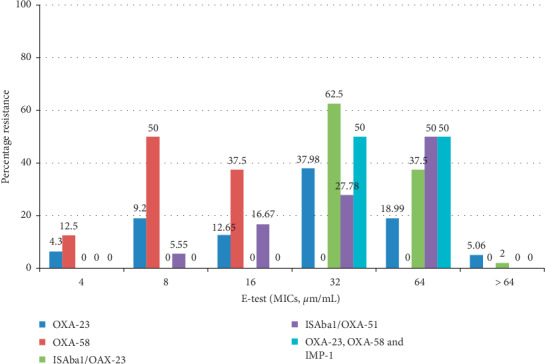
Distribution of imipenem MICs in 100 CRAB isolates with the *bla*_OXA-23-like_, *bla*_OXA-58-like_, IS*Aba*1*/bla*_OXA-23-like_, IS*Aba*1*/bla*_OXA-51-like_ and *bla*_OXA-23-like_, *bla*_OXA-58-like_ and *bla*_IMP-1_ genes.

**Figure 3:**
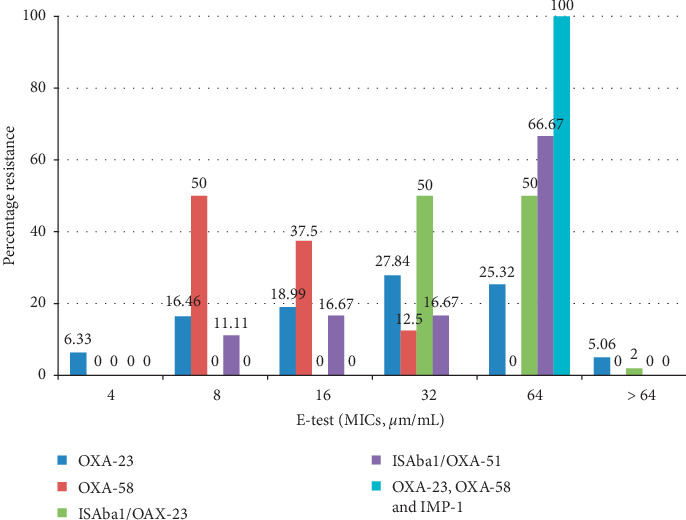
Distribution of meropenem MICs in 100 CRAB isolates with the *bla*_OXA-23-like_, *bla*_OXA-58-like_, IS*Aba*1*/bla*_OXA-23-like_, IS*Aba*1*/bla*_OXA-51-like_ and *bla*_OXA-23-like_, *bla*_OXA-58-like_ and *bla*_IMP-1_ genes.

Results showed that all of the strains investigated in this study had the intrinsic *bla*_OXA-51-like_ gene. Among them, 70% were positive for the *bla*_OXA-23-like_, 8% were positive for *bla*_OXA-58-like,_ and 5% positive for *bla*_OXA-24-like_. For the MBLs, 4% strains were positive for *bla*_IMP-1_, 3% carried *bla*_VIM_, 2% carried *bla*_NDM-1,_ and none of the tested isolates harboured the *bla*_SIM_. Alleles encoding *bla*_AmpC_ enzymes were not detected in any strain. The IS*Aba*1 was located upstream the *bla*_OXA-51-like_ and the *bla*_OXA-23-like_ carrying strains 15% and 40%, respectively. Some of the strains coharboured different carbapenemase genes mostly yielding very broad-spectrum antibiotic resistance profiles to imipenem and meropenem. Both strains possessing the IS*Aba*1/*bla*_OXA-23-like_ and *bla*_OXA-23-like_, *bla*_OXA-58-like,_ and *bla*_IMP-1_ genes had the highest carbapenem resistance with these strains having imipenem MICs of 32 *µ*g/mL or more, and having meropenem MICs of 32 *µ*g/mL or more. This was followed by the strains carrying the *bla*_OXA-23-like_ gene with 62% having imipenem MICs of 32 *µ*g/mL or more, and 58% having meropenem MICs of 32 *µ*g/mL or more (Figures 2 and 3). Screening for the presence of integron integrase genes showed the presence of the class 1 integrase gene in 80% MDR *A. baumannii* strains (Table 3).

## 4. Discussion

Understanding the fundamental mechanism that underlines *A. baumannii* infections, including original source of infecting strains, resistance patterns, and genes responsible for the development of resistance is critical for the development of appropriate infection control measures and more efficient treatment strategies [5]. Data from countries that lack routine surveillance, including South Africa, are necessary in order to understand and prevent the dissemination of carbapenem-resistant strains of species that are in epidemiological expansion, like *A. baumannii*.

Results from the present study revealed a 100% resistant rate of MDR *A. baumannii* to third-generation cephalosporins (ceftazidime, cefotaxime, and ceftriaxone). Resistance to cefepime was 87%. All strains were resistant to piperacillin, piperacillin/tazobactam, trimethoprim/sulfamethoxazole, and ampicillin/sulbactam. The percentage rates of resistance to remaining antibiotics range from 48% to 89% with the exception of colistin which had no resistant strain detected. Carbapenem resistance of *A. baumannii* is a major concern since it is the drug of choice in the treatment of *A. baumannii* infections [26]. The results showed that the majority of *A. baumannii* strains tested were resistant to both imipenem 81% and meropenem 83%. Lowings et al. found all MDR *A. baumannii* isolates showed high level resistance (cefotaxime 100%; cefotaxime 100%; ceftazidime 89%; cefepime 90%; imipenem 86%; and meropenem 86%) in a study conducted at the Department of Medical Microbiology, Prinshof Campus, University of Pretoria/National Health Laboratory Service (NHLS) in 2013 [27]. A previous study by Kock et al. in the same setting showed that the overall percentage of resistance to the cephalosporin antibiotics was cefepime (62%) and ceftazidime (45%) [28]. A report on antimicrobial resistance surveillance from sentinel public hospitals in South Africa in 2013 showed a resistance rate of 80% to cefepime and 73% to ceftazidime [29]. Comparing the results of the present study with these earlier studies indicates that the resistant rate of MDR *A. baumannii* to various antibiotics has not decreased. High levels of resistance to third and fourth generation cephalosporins in this study indicated that cephalosporins are no longer efficacious in the treatment of MDR *A. baumannii* isolated in South Africa. The full susceptibility of all MDR *A. baumannii* to colistin in this study indicated that colistin is still a better option of the drug for the treatment of infections caused by *A. baumannii* in South African hospitals. However, the issue of nephrotoxicity, neurotoxicity, colistin-resistance, and heteroresistance shown by colistin monotherapy is a challenge in the management of this infection [30]. The high resistance rate to other antibiotic categories may probably be due to the heavy selection pressure from overuse of these antibiotics.

Although *A. baumannii* infections are observed in all body parts, they are mostly observed in the respiratory system. Our study showed that *A. baumannii* isolates were predominantly found in the respiratory samples (tracheal aspirate) and the most frequent setting for MDR *A. baumannii* was found in the ICUs (41%). ICUs (Neonatal and general intensive care) are settings where most patients have indwelling devices and invasive procedures. Guckan et al. and Lowings et al. isolated 85.2% and 12% *A. baumannii* isolates, respectively, from ICUs [27, 31].

Molecular detection of carbapenemase-encoding genes has shown that the production of OXA-type carbapenemases is predominant in *A. baumannii* [30, 32]. The chromosomally encoded *bla*_OXA-51-like_ gene was detected in all the strains supporting those of other studies demonstrating that the detection of the *bla*_OXA-51-like_ gene can be used as a supplementary tool to identify *A. baumannii* at the species level together with additional methods [33]. It was observed that the expression of *bla*_OXA-23-like_ was the second dominant carbapenem resistance mechanism (70%) in MDR *A. baumannii* among the strains investigated aside the intrinsic *bla*_OXA-51-like_ gene. Its prevalence among isolates was higher than this in previous reports in South Africa. Its prevalence among strains was higher in this study than in the previous report (59%) by Kock et al., whilst Lowings et al. reported a prevalence of 77%. The *bla*_OXA-23-like_ gene is considered a virulence biomarker and a significant cause of carbapenem resistance worldwide [34]. This gene was first reported in 1985 in Scotland and since then outbreaks of OXA-23 carbapenemase-producing *A. baumannii* have been reported all over the world and is a dominant genetic determinant in the Asia subcontinent [35]. In Latin America, Brazil was the first country to report OXA-23-like-producing isolates in 2003, followed by Colombia, *Argentina*, and Mexico some years later [36].

This study also detected *bla*_OXA-24-like_ and *bla*_OXA-58-like_ genes in 5% and 8% isolates, respectively. Although the acquired *bla*_OXA-23-like_ is the dominant genetic determinant reported in most studies in South Africa, the detection of *bla*_OXA-58-like_ and *bla*_OXA-24-like_ genes in our study shows that these genes are becoming significant in carbapenem resistance in *A. baumannii* and may be responsible for the increase in carbapenem resistance in *A. baumannii.* It also suggested that these oxacillinases may locally spread in Mthatha, South Africa. These findings concur with a study by Ji et al. in China, who reported that *bla*_OXA-23-like_ are the most frequent carbapenem-resistant *A. baumannii*, whereas *bla*_OXA-24-like_ and *bla*_OXA-58-like_ genes pose potential threats of hospital outbreaks of multidrug-resistant *A. baumannii* [37]. For class B metallo-*β*-lactamases, we found that 4%, 3%, and 2% of the isolates had *bla*_IMP-1_, *bla*_VIM,_ and *bla*_NDM-1,_ respectively, and no isolate was found to produce *bla*_SIM_ gene. None of the clinical *A. baumannii* isolates harboured *bla*_AmpC_. IMP (primarily detected in South Korea) and NDM-1 (recently reported in many countries, such as India, Israel, Egypt, Germany, Spain, Switzerland, the United Arab Emirates, and China) are the two MBL genes most frequently detected in *A. baumannii* isolates [7, 33]. The *bla*_NDM-1_ producing bacteria are mainly reported in Enterobacteriaceae [38]. This gene was first identified from *Klebsiella pneumoniae* and *E. coli* strains from a patient previously hospitalized in India and has recently emerged in *A. baumannii* isolates [32]. Taking into consideration the diversity of the South African community (prominence of Asian and European communities where *bla*_NDM-I_ have been reported) and the frequency of travels made by individuals between these countries, it is believed that the spread of *bla*_NDM-1_ was likely to occur mostly through *A. baumannii* rather than Enterobacteriaceae. However, these findings need further investigation.

Interestingly, this study showed that some isolates were found to coharbour carbapenemase genes. Among these isolates include the coharbouring of carbapenemase genes *bla*_OXA-23-like_ and *bla*_IMP-1_ in 1% isolate, *bla*_OXA-23-like_ and *bla*_OXA-58-like_ in 2% isolates, and *bla*_OXA-23-like_, *bla*_OXA-58-like_, and *bla*_IMP-1_ in 2% isolates. To the best of our knowledge, this is the first report of the coharbouring of *bla*_OXA-23-like_, *bla*_OXA-58-like_, and *bla*_IMP-1_ in the Eastern Cape province of South Africa. We believe that coharbouring of these resistance genes is due to the presence of different genes in the same strain. This warrants the use of molecular typing methods to determine the clonal types of the isolates. The coharbouring of these major resistance mechanisms seriously limits therapeutic options, raising concerns regarding their transmission to other organisms. For example, we found that all isolates that coharboured *bla*_OXA-23-like_, *bla*_OXA-58-like,_ and *bla*_IMP-1_ had imipenem MICs of 32 *μ*g/mL or more and meropenem MICs of 64 *μ*g/mL.

The study showed that carbapenem susceptibility phenotypes of our *A. baumannii* isolates clearly correlated with the presence or absence, in the genomes, of IS*Aba*1-linked *bla*_OXA-23-like_ or *bla*_OXA-51-like_ genes (Figures 2 and 3). For instance, the 100% MDR isolates with IS*Aba*1 elements upstream of the *bla*_OXA-23-like_ had imipenem and meropenem MICs 32 *μ*g/mL or more, whereas 83.34 and 77.78% of MDR isolates with IS*Aba*1 elements upstream of the *bla*_OXA-51-like_ had imipenem and meropenem MICs 32 *μ*g/mL, respectively. Presumably, the IS*Aba*1 element likely provides exogenous promoter functions to overexpress the linked genes. Evidence showed that carbapenem resistance mediated by overexpression of *bla*_OXA-like_ families due to upstream IS elements is more prevalent [39–42]. Our data are consistent with the findings of previous studies in that acquisition IS*Aba*1 are the main mechanism of carbapenem resistance among *A. baumannii* by acting as a promoter to increase oxacillinase expression [9, 39, 43]. However, there are limited studies in South Africa as most of the studies evaluated the prevalence of OXA-like families in detail. There is, therefore, the need for further investigations to find the contribution of other IS elements (IS*Aba*2, IS*Aba*3) upstream to OXA-like families in different parts of the country. The results suggested that class 1 integrons did not contribute resistance to carbapenems since its presence did not have an effect on the MICs of carbapenems.

### 4.1. Limitations

The study has some limitations. Firstly, the study was conducted in a single centre, NMAH in the Tambo district municipality, so our findings may not be generalized to other settings. Secondly, the study did not include molecular typing epidemiological study reference methods such as PFGE, MLST, REP-PCR, etc. which would have better evaluated the dissemination of a carbapenemase or some clones to understand CRAB isolates in the clinical setting. These studies are necessary to help highlight the need for area-specific surveillance to inform appropriate management.

## 5. Conclusion

In conclusion, the results of this study provide direct evidence of the spread of carbapenemase-encoding genes especially *bla*_OXA-23-like_ in our setting. High presence of the IS*Aba*1 element upstream of the *bla*_OXA-51-like_ and *bla*_OXA-23-like_ increases expression of these carbapenemases and presents an emerging threat in this study setting. The results also provided clinically relevant insights into antimicrobial susceptibility and knowledge of the prevalent resistance mechanism among CRAB. This information will help in preventing the development of MDR *A. baumannii* in hospitals and also helps clinicians in the empirical treatment of patients. To prevent the development of Carbapenem-resistant *A. baumannii,* routine implementation of simple and cost-effective screening methods to detect carbapenemase production in hospitals is crucial in enhancing infection control practices and finally establishing antimicrobial stewardship programs. Contribution and interplay of different mechanisms to the rapid increase in antibiotic resistance of *A. baumannii* in South Africa warrant further investigation.

## Figures and Tables

**Table 1:** Quality control (QC) organisms used for the detection of resistant genes in this study.

Organism	ATCC or NCTC number	Resistant gene(s)
*A. baumannii*	NCTC 13302	OXA-26 (OXA-24-like; OXA-51-like
*A. baumannii*	NCTC 13305	OXA-58
*Klebsiella pneumoniae*	ATCC-BAA 2146	NDM
*A. baumannii*	NCTC 13301	OXA-23
*A. baumannii*	NCTC 13303	OXA-26 (OXA-51-like)
*A. baumannii*	NCTC 13304	OXA-27 (OXA-51-like)
*Enterobacter cloacae*	NCTC 13405	AmpC
*Pseudomonas aeruginosa*	NSCC stock	VIM
*A. baumannii*	ATCC 19606	OXA-51
*Escherichia coli*	NCTC 13476	IMP-1
*Escherichia coli*	Se 131 (accession number: AJ238350)	*intI*1
*Pseudomonas aeruginosa*	ATCC 27853	OXA-24-like; OXA-51-like
*Klebsiella pneumoniae*	ATCC 8303	SIM-1
*A. baumannii*	AYE strain	AmpC, IS*Aba*1, OXA-51-like

**Table 2:** List of primers used for PCR amplification of resistant genes.

Gene	Oligonucleotide sequence	Fragment size (bp)	Location	References
16SrRNA	F: 5′TTT AAG CGA GGA GGA GG3′ R: 5′ATT CTA CCA TCC TCT CCC3′	240	16*SrRNA*	[20]
OXA-23-like	F: 5′GATCGGATTGGAGAACCAGA3′ R: 5′ATTTCTGACCGCATTTCCAT3′	501	*bla*_OXA-23_	[21]
OXA-24-like	F: 5′GGTTAGTTG GCC CCC TTA AA3′ R: 5′AGTTGAGCGAAAAGGGGATT3′	246	*bla*_OXA-24_	[21]
OXA-51-like	F: 5′TAATGCTTT GATCGG CCT TG3′ R: 5′TGGATTGCACTT CAT CTT GG3′	353	*bla*_OXA-51_	[21]
OXA-58-like	F: 5′AAGTATTGGGGCTTGTGCTG-3′ R: 5′CCCCTCTGCGCTCTACATAC3′	599	*bla*_OXA-58_	[21]
IMP-1	F: 5′GATGGTATGGTGGCTCTTGT3′ R: 5′TTAATTTGCCGGACTTAGGC3′	448	*Bla*_IMP_	[22]
NDM-1	F: 5′ATTAGCCGCTGCATTGAT3′ R: 5′CATGTCGAGATAGGAAGTG3′	154	*bla*_NDM_	[23]
VIM-like	F: 5′ACTCACCCCCATGGAGTTTT3′ R: 5′ACGACTGAGCGATTTGTGTG3′	815	*bla*_VIM_	[24]
SIM-1-like	F: 5′TAATGCTTT GATCGG CCT TG3′ R: 5′TGGATTGCACTT CAT CTT GG3′	353	*bla*_SIM_	[21]
AmpC	F: ACAGAGGAGCTAATCATGCG R: GTTCTTTTAAACCATATACC	1243	*bla*_AmpC_	[25]
*ISAba*1	F: 5-CACGAATGCAGAAGTTG-3 R: 5-CGACGAATACTATGACAC-3	599	IS*Aba*1	[18]
*intI*1	F: 5′CAG TGG ACA TAA GCC TGT TC3′ R: 5′CCC GAC GCA TAG ACT GTA3′	160	Integrase gene	[19]

**Table 3:** Percentage distribution of carbapenemases-encoding genes in 100 CRAB strains.

Carbapenemases-encoding genes	Number (%)
*bla*_OXA-51-like_ only	0
*bla*_OXA-51-like_ and *bla*_OXA-23-like_	23
*bla*_OXA-51-like_ and *bla*_OXA-58-like_	4
*bla*_OXA-51-like_ and *bla*_OXA-24-like_	5
*bla*_OXA-51-like_ and *bla*_IMP-1_	1
*bla*_OXA-51-like_ and *bla*_VIM_	3
*bla*_OXA-51-like_ and *bla*_NDM-1_	2
IS*Aba*1*/bla*_OXA-51-like_	15
*bla*_OXA-51-like_ and IS*Aba*1*/bla*_OXA23-like_	40
*bla*_OXA-51-like_ and *bla*_OXA23-like_ and *bla*_IMP-1_	1
*bla*_OXA-51-like_ and *bla*_OXA-23-like_ and *bla*_OXA-58-like_	2
*bla*_OXA-51-like_ and *bla*_OXA-23-like_ and *bla*_OXA-58-like_ and *bla*_IMP-1_	2
IS*Aba*1/*bla*_OXA-51-like_ and IS*Aba*1/*bla*_OXA-23-like_	2
*intI*1 only	0

## Data Availability

All data generated or analyzed during this study are included in this published article (and its supplementary information file).

## Supplementary Materials

Supplementary Materials Details of the genotypic features of *Acinetobacter baumannii* isolates consisting of genes encoding Amber class D serine-carbapenemase genes (blaOXA-51-like, blaOXA-23-like, blaOXA-24-like, and blaOXA-58-like), Amber class B metallo-*β*-lactamases (blaIMP-1, blaVIM, blaSIM, and blaNDM-1) and Amber class C blaAmpC. Age range, gender, ward, and isolate source are also indicated.
