# Supplementary material for: Molecular Detection of Carbapenemase-Encoding Genes in Multidrug-Resistant Acinetobacter baumannii Clinical Isolates in South Africa
Source: Int J Microbiol. 2020 Jun 13;2020:7380740. doi: 10.1155/2020/7380740 (PMC7306865; doi:10.1155/2020/7380740)
Supplement: Supplementary Materials — Details of the genotypic features of Acinetobacter baumannii isolates consisting of genes encoding Amber class D serine-carbapenemase genes (blaOXA-51-like, blaOXA-23-like, blaOXA-24-like, and blaOXA-58-like), Amber class B metallo-β-lactamases (blaIMP-1, blaVIM, blaSIM, and blaNDM-1) and Amber class C blaAmpC. Age range, gender, ward, and isolate source are also indicated. [file 7380740.f1.docx]

**SUPPLEMENTARY MATERIAL**

**Genotypic features of 100 *A. baumannii* clinical isolates collected from NMAH from August 2016-July 2017.**

| Age range | Gender (M) (F) | Ward (n) | Isolate source (n) | *bla*_OXA-23_ | *bla*_OXA-24_ | *bla*_OXA-58_ | *bla*_IMP-1_ | *bla*_NDM-1_ | *bla* _VIM_ | *bla* _SIM_ | *bla*_AmpC_ | IS*Aba1* | *intI1* |
| --- | --- | --- | --- | --- | --- | --- | --- | --- | --- | --- | --- | --- | --- |
| 0-9 | (12) (17) | ICU (3)  SW (2)  NW (22)  GW (1)  MW (1) | P (2)  BLD (5)  TA (12)  CT (5)  CSF (0)  SPM (3)  URN (2) | 21 | 2 | 3 | 1 | 1 | 1 | - | - | 20 | 14 |
| 10-19 | (5) (4) | ICU (5)  SW (1)  GW (2)  MW (1) | P (0)  BLD (4)  TA (1)  CT (0)  CSF (1)  SPM (0)  URN (3) | 5 | 1 | - | - | - | - | - | - | 4 | 1 |
| 20-29 | (2) (5) | ICU (4)  SW (1)  GW (1)  MW (1) | P (1)  BLD (2)  TA (2)  CT (0)  CSF (0)  SPM (1)  URN (1) | 3 | - | 1 | - | - | - | - | - | 5 | 3 |
| 30-39 | (3) (2) | ICU (3)  SW (1)  GW (0)  MW (1) | P (0)  BLD (1)  TA (2)  CT (1)  CSF (0)  SPM (1)  URN (0) | 4 | - | - | - | - | - | - | - | 1 | 3 |
| 40-49 | (3) (0) | ICU (1)  SW (1)  GW (0)  MW (1) | P (0)  BLD (2)  TA (1)  CT (0)  CSF (0)  SPM (0)  URN (0) | 2 | - | - | - | - | - | - | - | 2 | - |
| 50-59 | (2) (2) | ICU (2)  SW (0)  GW (1)  MW (1) | P (1)  BLD (1)  TA (1)  CT (0)  CSF (0)  SPM (1)  URN (0) | 4 | - | - | - | - | - | - | - | 4 | 3 |
| 60-69 | (2) (6) | ICU (4)  SW (2)  GW (1)  MW (1) | P (1)  BLD (2)  TA (3)  CT (0)  CSF (1)  SPM (1)  URN (0) | 5 | - | - | - | - | - | - | - | 7 | 5 |
| 70-79 | (5) (2) | ICU (1)  SW (3)  GW (1)  MW (2) | P (1)  BLD (2)  TA (1)  CT (0)  CSF (0)  SPM (1)  URN (2) | 4 | 1 | 1 | - | - | 1 | - | - | 7 | 5 |
| 80-89 | (4) (7) | ICU (6)  SW (2)  GW (2)  MW (1) | P (1)  BLD (2)  TA (5)  CT (1)  CSF (0)  SPM (2)  URN (0) | 9 | - | 1 | 1 | 1 | 1 | - | - | 3 | 2 |
| 90-upwards | (9) (8) | ICU (12)  SW (2)  GW (0)  MW (3) | P (4)  BLD (3)  TA (4)  CT (0)  CSF (0)  SPM (4)  URN (2) | 14 | 1 | 2 | 2 | - | - | - | - | 4 | 9 |

Abbreviations: **CT**, Catheter tip; **ICU**, intensive care unit; **TA**, Tracheal aspirate; **WS**, Wound swab; **SPM**, Sputum; **BLD**, Blood; **P**, Pus; **CSF** Cerebrospinal fluid; **URN,** Urine; **NW**, Neonatal ward; **SW**, Surgical ward, **MW**, Medical ward; **GW**, General ward; **M,** Male; **F**, Female
